# Identification and analysis of driving factors for ecosystem service bundles in Shanxi Province under multiple scenario simulations

**DOI:** 10.1038/s41598-025-08876-5

**Published:** 2025-07-01

**Authors:** Guofeng Dang, Guibin Li, Jinzhou Hu

**Affiliations:** School of Geography and Environmental Sciences, Northwest Normal University, Lanzhou, 730070 China

**Keywords:** Land utilization, Ecosystem service bundles, Evolutionary trajectory, Multiple scenario simulation, Shanxi Province, Ecological modelling, Ecosystem ecology, Ecosystem services

## Abstract

In the context of carbon peak and energy structure transformation, ecosystem service bundle(ESB) have obvious changes. As a typical ecologically fragile area in China, the study of ESB in Shanxi Province plays a significant effect in regional sustainable development and ecological governance. This paper employs the PLUS model to simulate land use patterns, which utilizes land use data about Shanxi Province for the years 1980, 2000, and 2020. By integrating this with the dynamic ecosystem service value (ESV) model to assess the ESV, the evolutionary trajectory of the ESB is systematically revealed. Additionally, the driving factors behind the changes in ESB are analyzed using geographic detectors. The results indicated that: (1)From 1980 to 2020, the area of cultivated land consistently decreased, while the area of construction land expanded rapidly. By 2040, the area of cultivated land under the NDS is projected to decrease by 4.21%, whereas under the FPS, it is expected to increase by 4.35% due to policy intervention. (2)The total value of ESV exhibited fluctuations in an ‘N-type’ pattern. From 1980 to 2020, there was an overall decline of 2.05%, but the ESV is projected to rebound by 0.84% in 2040(FPS). (3)The synergistic relationship among ecosystem services was dominant, accounting for 88.79%, yet the trade-off coefficient between FP-CR increased by 23.5% over the past decade, which is underscoring the significant conflict between food production and ecological protection.(4)Three types of ESBs were identified: the agricultural production-leading bundle (ESB1), the ecological regulation-strengthening bundle (ESB2), and the water conservation-sensitive bundle (ESB3).The proportion of stable types reached its peak at 82.91% under the AEDS, highlighting the reinforcing effect of market mechanisms on ecological function lock-in. The research findings can provide valuable decision support for land space optimization in ecologically fragile areas and the value transformation of ecological product.

**Supplementary Information:**

The online version contains supplementary material available at 10.1038/s41598-025-08876-5.

## Introduction

Ecosystem services (ES) refer to the material and non-material benefits that human society directly or indirectly obtains from the structure, process and function of the ecosystem system^1^.It is the cornerstone of maintaining human well-being and socio-economic development^2^.It plays an irreplaceable role in maintaining ecological integrity, ensuring food security, purifying water bodies and regulating climate^3^. Rapid urbanization and intensive land use have seriously disrupted the level and capacity of ecosystem services^4^. Ecosystem Services Value (ESV) is an indicator to quantify ecosystem services and measure the level of ecosystem services. Assessing the value of ecosystem services helps to weigh the pros and cons in the decision-making process and promotes the protection and management of natural resources^5^. Strengthening the research on the changing trend of ecosystem service value and its driving factors on the spatial and temporal scales, and exploring its relationship with natural environment and socio-economic factors^6^ provide a strong theoretical basis for maintaining and repairing ecosystem service capacity, enhancing and optimizing ecosystem service value, exploring and realizing the “ win-win” or “ multi-win ” goals such as trade-offs and synergies of multiple ecosystem services. It is crucial to maintain the coordination and cooperation of regional ecosystems and is the key to the continuous functioning of ecosystem service functions^7^. Ecosystem Service Bundles (ESB), which is a collection of ecosystem services with similar spatial and temporal differentiation characteristics, provides a new perspective for integrated ecosystem management^8^, and provides new theoretical support and practical guidance for ecological governance and sustainable development strategies.

ESB research can provide scientific support for ecological protection priority zoning and sustainable land use planning by identifying dominant service composition and its dynamic evolution^9^. Although significant progress has been made in the field of ecosystem service value quantification^10^ and spatial mapping^11^, the research on the evolution mechanism of ESB under multi-scenario simulation is still weak^12^. The Loess Plateau region of China is particularly typical: the increasing level of urbanization has exacerbated land use conflicts and led to the degradation of ecosystem service functions^13^. In recent years, the study of ecosystem service clusters has received extensive attention. Zhou Shihao et al.studied the ecosystem services cluster of the Yangtze River Delta by constructing a global climate change scenario and a regional policy-oriented local scenario group^14^; Li Huiqiang et al.studied the changes and driving forces of ecosystem service clusters in Guantian Economic Zone from multiple time series^15^; He Guoyu et al. evaluated the ecological risk and influencing factors by studying the supply and demand cluster of ecosystem services in Wuhan metropolitan area, and emphasized that the supply and demand cluster of ecosystem services should be included in the ecological risk study^16^; Chen Tianqian et al. found that the distribution of clusters was strongly disturbed by human socio-economic behavior through the study of ecosystem service clusters in Beijing-level surrounding areas, which provided a reference for ecosystem service management policies in Beijing and its surrounding areas^17^; Jiang Hongbo et al.studied the spatial and temporal evolution of the ecosystem service cluster of the Beibu Gulf urban agglomeration in China, and provided valuable opinions for understanding the complex relationship between human activities and the natural process of landscape formation^18^; Cheng Xin et al.analyzed the bundles, trade-offs and synergies of cultural ecosystem services (CES) in Huanhuaxi Park through questionnaires, participatory mapping and interviews^19^. At present, the research on ecosystem services is deepening, and the research methods and models are also developing. The research not only focuses on the quantitative assessment of ecosystem services^20^, but also involves functional relationships^21^ and optimal management^22^.At the same time, the research is also exploring the spatial and temporal variability of ecosystem services^23^, as well as the trade-offs and synergies between ecosystem services^24^, which provides a better research method for the study of ecosystem service bundles. However, systematic research on the dynamic evolution of ESB under multi-scenario paths is still scarce. The existing research results mostly focus on single service value evaluation or static spatial pattern analysis, and lack the exploration of ESB spatio-temporal evolution mechanism based on multi-scenario simulation.

Located in the middle reaches of the Yellow River Basin, Shanxi Province is a typical region where resource-based economy and fragile ecology coexist. Diverse ecosystems such as forests, grasslands, farmlands and wetlands are developed in the region^25^, which bear important ecological functions such as food production, soil conservation, and air purification^26^. However, with the rapid advancement of industrialization and urbanization, the province’s ecosystem is facing unprecedented challenges. Problems such as reduced arable land resources, water shortages, and environmental pollution have seriously affected ecosystem services^27^.

**Fig. 1:**
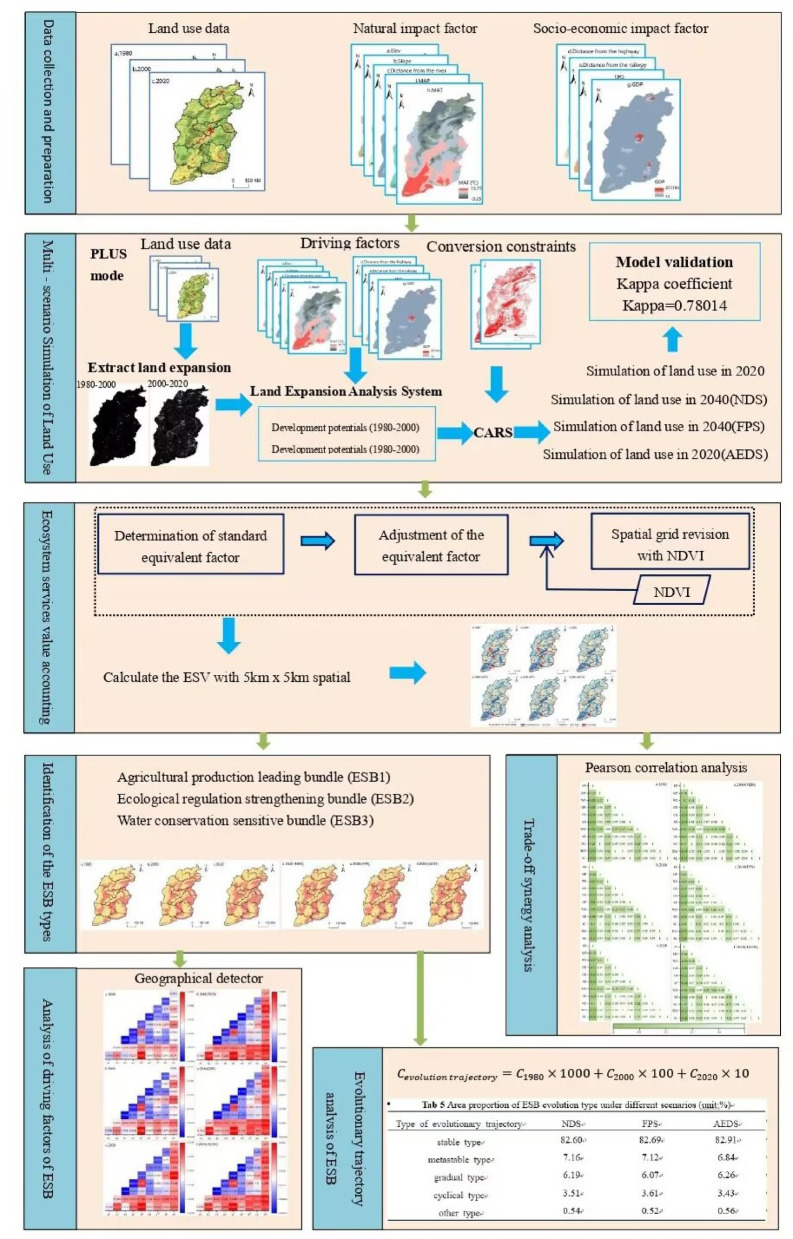
Study framework.

In this study, the PLUS model and the dynamic ecosystem service value accounting system were used to systematically analyze the dynamic changes and driving mechanisms of ESB in Shanxi Province in 1980, 2000, 2020 and 2040 under three scenarios of Natural development, Farmland Protection and Accelerated Economic Development Scenario (Fig. 1**)**.

## Research methods and data sources

### Overview of the study area

**Fig. 2:**
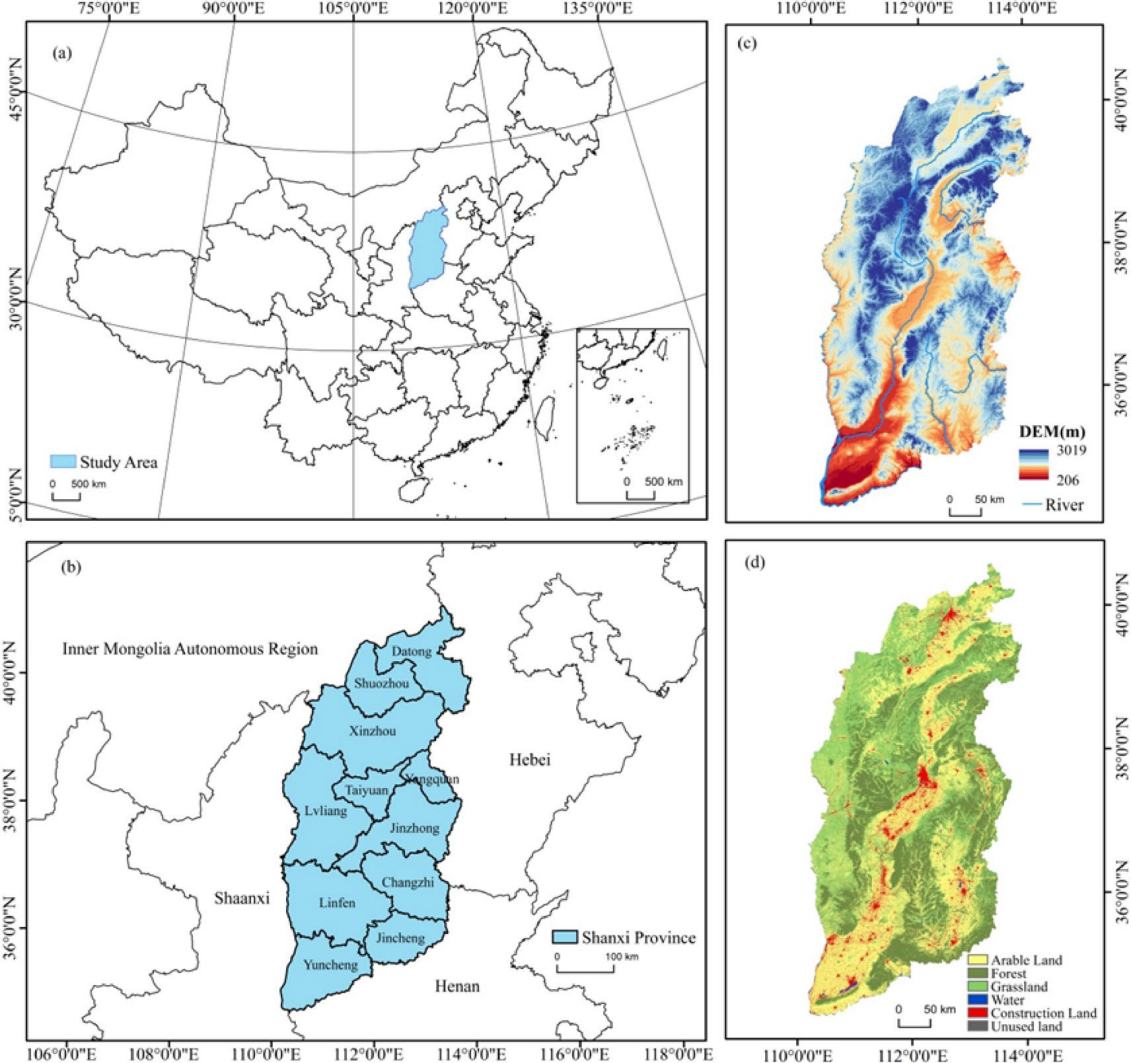
Study area.

Shanxi Province is located in the eastern part of the Loess Plateau in North China. The geographical coordinates are between 34°34′*N* ~ 40°44′N, 110°14′E ~ 114°33′E. It is adjacent to Hebei Province in the east, Shaanxi Province in the west, Henan Province in the south, and Inner Mongolia Autonomous Region in the north (Fig. 2). The total area is about 156,700 square kilometers. As an important energy and heavy industry supply base in China, Shanxi Province is known as the coal sea^28^.

The terrain of Shanxi Province generally presents a pattern of “two mountains and one river”. The eastern part is the Taihang Mountains, the western part is the Lvliang Mountains, and the central part is the Xinding, Taiyuan, Linfen, Yuncheng and other beaded basins. The altitude gradually decreases from east to west, forming a typical loess plateau landform. The mountains and hills in the territory account for more than 80% of the total area. The surface is broken, the gullies are vertical and horizontal, and the problem of soil erosion is prominent^29^. The climate type belongs to temperate continental monsoon climate, with four distinct seasons, average annual temperature of 9 ~ 11 °C, and annual precipitation of 400 ~ 650 mm.

As of 2023, the resident population of Shanxi Province is 34,659,900, the total GDP is 25,698.18 billion yuan, and the per capita GDP is about 73,984 yuan, of which the primary industry is 138.886 billion yuan, accounting for 5.4%; the secondary industry was 1332.969 billion yuan, accounting for 51.9%; the tertiary industry is 10979.64 billion yuan, accounting for 42.7%^30^.

Shanxi Province is a key area for ecological protection in the Yellow River Basin. Its tributaries such as the Fen River provide water supply for the Yellow River, and ecological protection projects ensure the water quality of the Yellow River; its ecosystem service function is remarkable, biodiversity is rich, and ecological security barrier function is outstanding. It is a demonstration site for ecosystem restoration and green transformation, and the traditional energy base realizes the transformation to green development^31^.

### Data source

#### Data source

The data used in this paper include land use data, meteorological data, socio-economic data, terrain data and other data (Table 1).

**Table 1:** Data type and source.

Data type	Formal	Temporal reference	Data sources
Land use data	30 m x 30 m	1980/2000/2020	http://www.resdc.cn/
DEM data	30 m x 30 m	2023	http://www.gscloud.cn/
NDVI	1 km x 1 km	1980/2000/2020/2024	http://www.gscloud.cn/
Roads, railroads, rivers		2023	http://www.webmap.cn/
Demographic data	1 km x 1 km	2020	http://tjj.shanxi.gov.cn/tjsj/tjnj/
average annual temperature	1 km x 1 km	2023	http://data.cma.cn/
Annual precipitation	1 km x 1 km	2023	http://data.cma.cn/
Other data	Statistical data	2023	http://tjj.shanxi.gov.cn/tjsj/tjnj/

#### Data preprocessing

Based on the 30 m resolution land use raster data provided by the resource and environment data platform of the Chinese Academy of Sciences, the original two-level classification system (25 categories) was merged into six categories: cultivated land, forest land, grassland, water area, construction land and unused land by using Arc Geographic Information System (ArcGIS) reclassification tool. Using Euclidean distance for roads, railways and rivers to generate distance maps from roads, railways and rivers; then use the vector boundary of Shanxi Province to extract the layers within Shanxi Province according to the mask; finally, the spatial reference coordinate system (WGS-1984) and the projection coordinate system (Asia _ North _ Albers _ Equal _ Area _ Conic) are uniformly established, and the resampling tool bilinear interpolation method is used to resample the non-30 m resolution data to the unified grid. The attribute association of vector-grid data is realized by spatial connection tool. ArcGIS10.5 version used in this article. (https://pan.baidu.com/s/10YxtXRUBZGa04F_B1ScoLQ?pwd=6789).

### Research methods

#### Patch-Generating land use simulation (PLUS) model

The Patch-Generating Land Use Simulation (PLUS) model was developed by the High Performance Spatial Computing Intelligent Laboratory of the College of Geography and Information Engineering of China University of Geosciences & National GIS Engineering Technology Research Center^13^. It mainly includes LEAS (Land Expansion Analysis System) module and CARS (Conversion of Agriculture, Rural Settlements) module. PLUS v1.40 version used in this article (https://github.com/HPSCIL/Patch - generating - Land - Use - Simulation - Model).

The LEAS module can extract and sample the land expansion part between the two periods of land use change, and use the random forest algorithm to mine and obtain the contribution rate and development probability of the driving factors of various types of land use. The CARS module combines random seed generation, transition transfer matrix and threshold decreasing mechanism to simulate future land use under the constraint of development probability. At the same time, the PLUS model also added Markov Chain for land use demand prediction and Kappa and Fom to verify the accuracy of the model^32^.

The domain weight parameter reflects the expansion intensity of each land use type, and the calculation formula is as follows:

1 $$\:{X}_{i}=\frac{{TA}_{i}-{TA}_{min}}{{TA}_{\text{m}\text{a}\text{x}}+{TA}_{min}}$$

Where: $\:{X}_{i}$ is the domain weight parameter of land use type $\:i$;$\:\:{TA}_{i}$ represents the expansion area of land use type $\:i$;$\:\:{TA}_{min}$ represents the minimum expansion area of various land use types;$\:\:{TA}_{max}$ represents the maximum expansion area of each land use type.

#### Dynamic ecosystem service value model

The ecosystem service equivalents for various land categories were determined using the Chinese terrestrial ecosystem service equivalents table established by Xie et al.^33^. The specific methodologies employed for these calculations are outlined as follows:

2 $$\:ESV=\sum\:_{\text{i=1}}^{\text{n}}{\text{A}}_{\text{i}}{\times\:VC}_{i}$$

Where: *ESV* refers to the value of the ecosystem service of the research area (yuan); *A*_*i*_ is the area of ​​the type *i* land type (hm^2^); the *VC*_*i*_ is the unit area of ​​the type *i* -type unit area of ​​the unit area of ​​the type *i* (yuan/hm^2^).

In reference to the studies conducted by Wang et al.^34^ and Gao et al.^35^, an appropriate formula was employed to adjust the value equivalent of the secondary ecosystem services present in Shanxi Province, taking into account the local context.

3 $$\:{E}_{a}=Q\times\:\:\frac{1}{7}F$$

4 $$\:{VC}_{i}={E}_{a}\times\:V$$.

Where: *E*_*a*_ represents the unit value equivalent (yuan/hm²); *Q* denotes the average grain output per unit area in the study area from 1980 to 2020 (kg/hm²); *F* indicates the average grain purchase price in the study area during the same period; and *V* refers to the equivalent value of different land types.

By introducing Normalized Difference Vegetation Index (NDVI) as the correction coefficient, the traditional equivalent factor method is dynamically adjusted. This improved method solves the limitations of traditional static equivalent factors in the dynamic changes of vegetation cover, and improves the spatial and temporal accuracy of ecosystem service value assessment, especially in the quantification of high vegetation cover areas such as forests and grasslands.

5 $$\:{F}_{K}=\frac{{NDVI}_{K}}{\stackrel{-}{NDVI}}$$

Where: *F*_*k*_ is the vegetation correction coefficient; *NDVI*_*k*_ is the average value of NDVI in the grid k; $\:\stackrel{-}{NDVI\:}$ is the average value of NDVI in the study area.

The calculation formula of dynamic ecosystem service value is as follows:

6 $$\:ESV=\sum\:_{\text{i=1}}^{\text{n}}{\text{A}}_{\text{i}}{\times\:{F}_{k}\times\:VC}_{i}$$

Where: *ESV* refers to the value of the ecosystem service of the research area (yuan); *A*_*i*_ is the area of ​​the type *i* land type (hm^2^); *F*_*k*_ is the vegetation correction coefficient; the *VC*_*i*_ is the unit area of ​​the type *i*-type unit area of ​​the unit area of ​​the type *i* (yuan/hm^2^).

The calculation formula of the contribution of land use change to ecosystem service value is as follows:

7 $$\:\Delta{ESV}_{i}=\sum\:_{i=1}^{n}{A}_{i}\times\:{F}_{k}\times\:\left(V{C}_{{i}_{after}}-V{C}_{{i}_{before}}\right)$$

8 $$\:C=\frac{\Delta{ESV}_{i}}{\Delta ESV}\times\:100\%$$

Where: $\:\Delta{ESV}_{i}$refers to the change of ecosystem service value (yuan); *A*_*i*_ is the area of ​​the type *i* land type (hm^2^); *F*_*k*_ is the vegetation correction coefficient; $\:V{C}_{{i}_{after}}$is the ecosystem service value per unit area (yuan/hm^2^) after the transformation of land use type $\:\text{i}$;$\:\:\text{V}{\text{C}}_{{\text{i}}_{\text{b}\text{e}\text{f}\text{o}\text{r}\text{e}}}$ is the ecosystem service value per unit area (yuan/hm^2^) before the change of land use type of type $\:\text{i}$; *C* is the contribution of land use change to the ecosystem services value;$\:\Delta{ESV}$ is the total variation of ecosystem service value.

#### Pearson correlation analysis

Pearson Correlation Coefficient is a widely used correlation measure in statistics to measure the strength and direction of the linear relationship between two continuous variables. It evaluates whether there is a significant linear correlation between variables by quantifying the degree of covariation between the two variables, and whether the nature of this correlation is positive or negative^36^. The formula is as follows:

9 $$\:r=\frac{\sum\:({\text{X}}_{\text{i}}-\stackrel{-}{\text{X}})({\text{Y}}_{\text{i}}-\stackrel{-}{\text{Y}})}{\sqrt{\sum\:{({\text{X}}_{\text{i}}-\stackrel{-}{\text{X}})}^{2}\sum\:{({\text{Y}}_{\text{i}}-\stackrel{-}{\text{Y}})}^{2}}}$$

Where: $\:{X}_{i}$and $\:{Y}_{i}$are the observed values of the two variables; $\:\stackrel{-}{X}$and $\:\stackrel{-}{Y}$are the sample mean values.

#### Elbow method

Elbow method is a method for determining the optimal number of bundles. It achieves this goal by analyzing the Within-Cluster Sum of Squares (WCSS) under different clustering numbers. The core idea is that as the number of bundles increases, WCSS usually decreases gradually. This is because more bundles mean that the distribution of data points in each bundle is more concentrated. However, when the number of bundles increases to a certain critical point, the decline rate of WCSS will obviously slow down. This turning point is called “elbow”, and the corresponding number of clusters is considered to be the best number of bundles^37^.

10 $$\:wcss\left(k\right)=\sum\:_{i=1}^{k}\sum\:_{x\in\:{C}_{i}}\left|\right|x-{u}_{i}|{|}^{2}$$

Where: *wcss (k)* is the sum of squares in the cluster under the cluster number k; *k* is the cluster number; $\:{C}_{i}\:$is the $\:i$th bundle; $\:{u}_{i}$ is the centroid of the bundle; *x* is the data point.

#### Evolutionary trajectory analysis of ecosystem service bundles

The evolution trajectory can be expressed as a map unit composed of the cluster types of the time phase in chronological order^38,39^. In this paper, three ecosystem service bundles are identified, and there are four periods in each time line. Therefore, there are 81 types of evolution trajectories in theory in each time line. The three ecosystem service clusters are coded as 1, 2, and 3 in turn. Using the grid calculator in ArcGIS software, the evolution trajectory coding is calculated by the following formula:

11 $$\:{C}_{evolution\:trajectory}={C}_{1980}\times\:1000+{C}_{2000}\times\:100+{C}_{2020}\times\:10+{C}_{2040}$$

Where: *C*_*evolution trajectory*_ is the evolution trajectory encoding; *C*_*1980*_, *C*_*2020*_, *C*_*2020*_, and *C*_*2040*_ correspond to the ecosystem service bundles type codes for the map unit in their respective years.

#### Geodetector

The core idea of the Geodetector is to decompose a geographical phenomenon into multiple sub-factors, analyze the correlation between these sub-factors and environmental factors one by one, and then clarify the influence intensity of each factor on the geographical phenomenon and its spatial distribution characteristics. Geodetector is composed of four sub-parts: risk detector, factor detector, ecological detector and interactive detector. It is a new statistical method to detect spatial heterogeneity and reveal the driving factors behind it^40^.

(1) Factor detection.

The spatial differentiation degree of a single factor X to the dependent variable Y is usually represented by q value. The calculation formula is:

12 $$\:q=\frac{{\sum\:}_{\text{h}=1}^{\text{L}}{\text{N}}_{\text{h}}{{\upsigma\:}}_{\text{h}}^{2}}{N{\sigma\:}^{2}}$$

Where: *q* is the strength of explanatory power; *L*is the classification number of dependent variables and independent variables; *h* is the number of driving factor layers; *N* and *N*_*h*_ are the number of samples of global and each classification h, respectively; *σ*^*2*^ is the variance of ESB.

(2) Interaction detection.

Interaction detection can identify the interaction of different driving factors on the dependent variable, that is, whether the evaluation factors X1 and X2 will increase or decrease the explanatory power of the dependent variable Y, or the influence of these factors on Y is independent of each other. There are five results of the interaction between the two independent variables on the dependent variable.(Table 2).

**Table 2:** Result types of two-factor interactions.

Judgment basis	Interaction type
q(X1∩X2) < Min(q(X1),q(X2))	Nonlinear weakening
Min(q(X1),q(X2)) < q(X1∩X2)< Max(q(X1),q(X2))	Single factor nonlinear weakening
q(X1∩X2) > Max(q(X1),q(X2))	Two-factor enhancement
q(X1∩X2) = q(X1) + q(X2)	independence
q(X1∩X2) > q(X1) + q(X2)	Nonlinear enhancement

## Results

### Multi-scenario simulation of land-use change

#### Scenario simulation settings

**Fig. 3:**
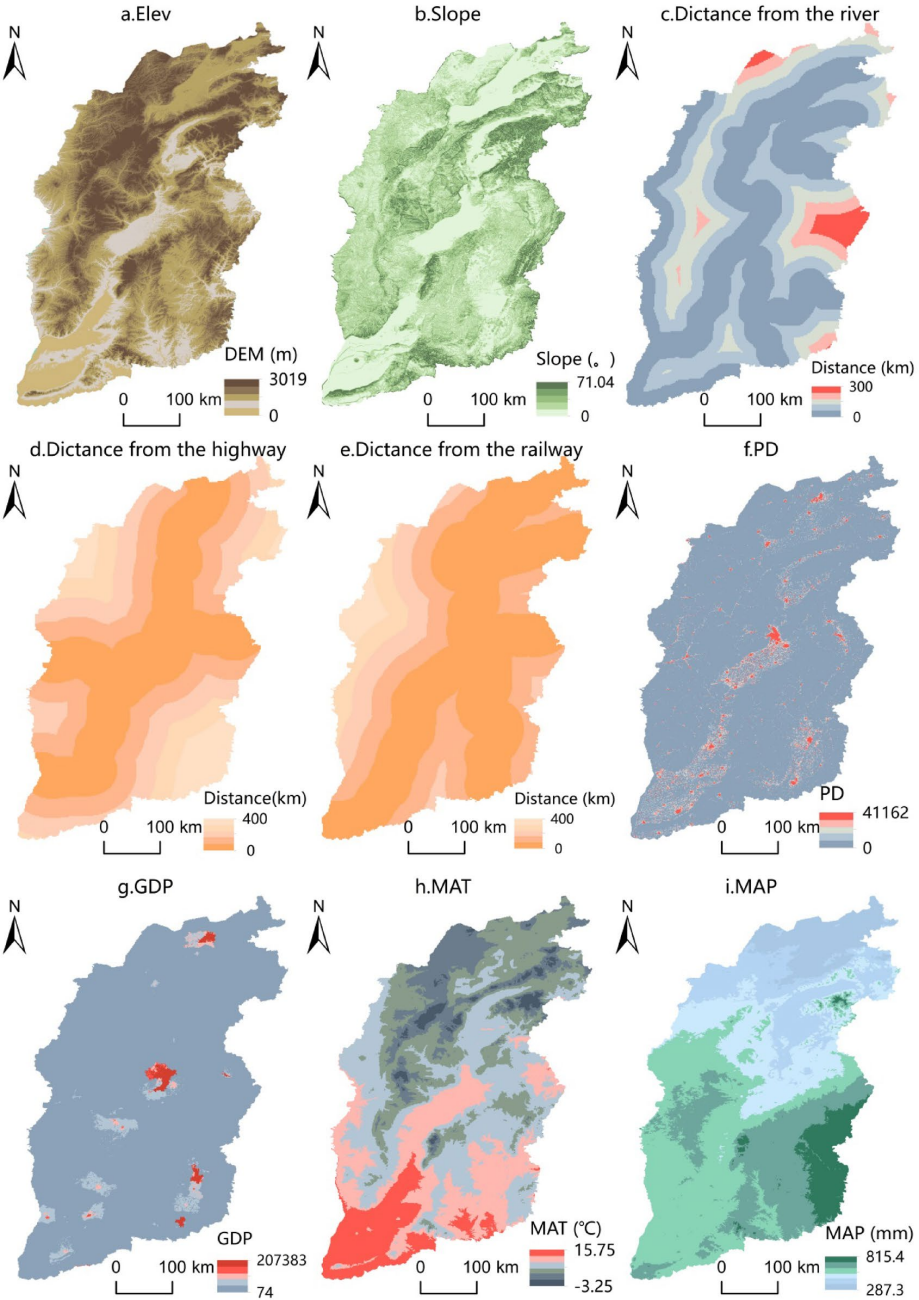
Atlas of restrictive conversion factors.

In this paper, 5 natural factors (elevation, slope, distance from river, average annual temperature and annual precipitation) and 4 socio-economic factors (distance from railway, distance from highway, population density and GDP) are selected to simulate land use (Fig. 3).

The predicted land use types in 2020 were compared with the actual land use types in 2020, and the Kappa value was calculated. The Kappa coefficient is 0.78014, and the overall accuracy is 84.82%, which meets the research requirements.

In order to comprehensively evaluate the impact mechanism of land use change on ecosystem services in Shanxi Province, this study constructed three simulation scenarios based on the PLUS model: Natural Development Scenario, Farmland Protection Scenario and Accelerated Economic Development Scenario. The scenario setting follows the progressive logic of “benchmark control-policy intervention-extreme development”, which not only reflects the historical inertia path.

of regional development, but also incorporates the differentiated orientation of national strategy and local planning, forming a multi-dimensional analytical framework for land use change(Table 3).

Scenario 1: Natural Development Scenario (NDS).

This scenario aims to simulate the natural succession process of land use without external intervention, focusing on the interaction between arable land, grassland and construction land in the process of urbanization. According to the principles of “respecting the laws of nature” in the *“Yellow River Basin Ecological Protection and High-quality Development Plan”*, as well as the strict protection requirements of the “five-water comprehensive reform” policy in Shanxi Province for water areas^41^. Based on the law of land use transfer from 1980 to 2020, the future land demand is predicted by Markov chain, and the water area is set as the restrictive land type to exclude its unnatural transformation to other land types. This scenario provides a baseline reference for subsequent analysis and reveals the spontaneous evolution trend of ecosystem services under the existing policy framework^42^.

**Table 3:** The probability matrix of different types of land transformation under different scenarios.

Scenario setting		AL	FO	GL	WA	CL	UL
NDS	AL	0.833050	0.031947	0.069884	0.004447	0.060514	0.000158
	FO	0.032169	0.914079	0.044315	0.001155	0.008168	0.000114
	GL	0.094806	0.052441	0.829667	0.001864	0.020996	0.000225
	WA	0.190143	0.030562	0.045479	0.655013	0.07346	0.005342
	CL	0.149377	0.013827	0.016756	0.003021	0.816643	0.000377
	UL	0.254614	0.033805	0.088867	0.062175	0.094781	0.465759
FPS	AL	0.870611	0.033387	0.073035	0.004648	0.018154	0.000165
	FO	0.032169	0.914079	0.044315	0.001155	0.008168	0.000114
	GL	0.094806	0.052441	0.829667	0.001864	0.020996	0.000225
	WA	0.190143	0.030562	0.045479	0.655013	0.073460	0.005342
	CL	0.194190	0.013099	0.015873	0.002862	0.773619	0.000357
	UL	0.381921	0.028031	0.073689	0.051556	0.078593	0.386210
AEDS	AL	0.833050	0.031947	0.069884	0.004447	0.060514	0.000158
	FO	0.032090	0.911820	0.044205	0.001152	0.010618	0.000114
	GL	0.094196	0.052104	0.824330	0.001852	0.027295	0.000224
	WA	0.190143	0.030562	0.045479	0.655013	0.073460	0.005342
	CL	0.089626	0.008296	0.010054	0.001813	0.889800	0.000411
	UL	0.241284	0.032035	0.084214	0.058920	0.142172	0.441375

Note: AL: Arable Land FO: Forest GL: Grass Land WA: Water CL: Construction Land UL: Unused Land.

Scenario 2: Farmland Protection Scenario (FPS).

**Fig. 4:**
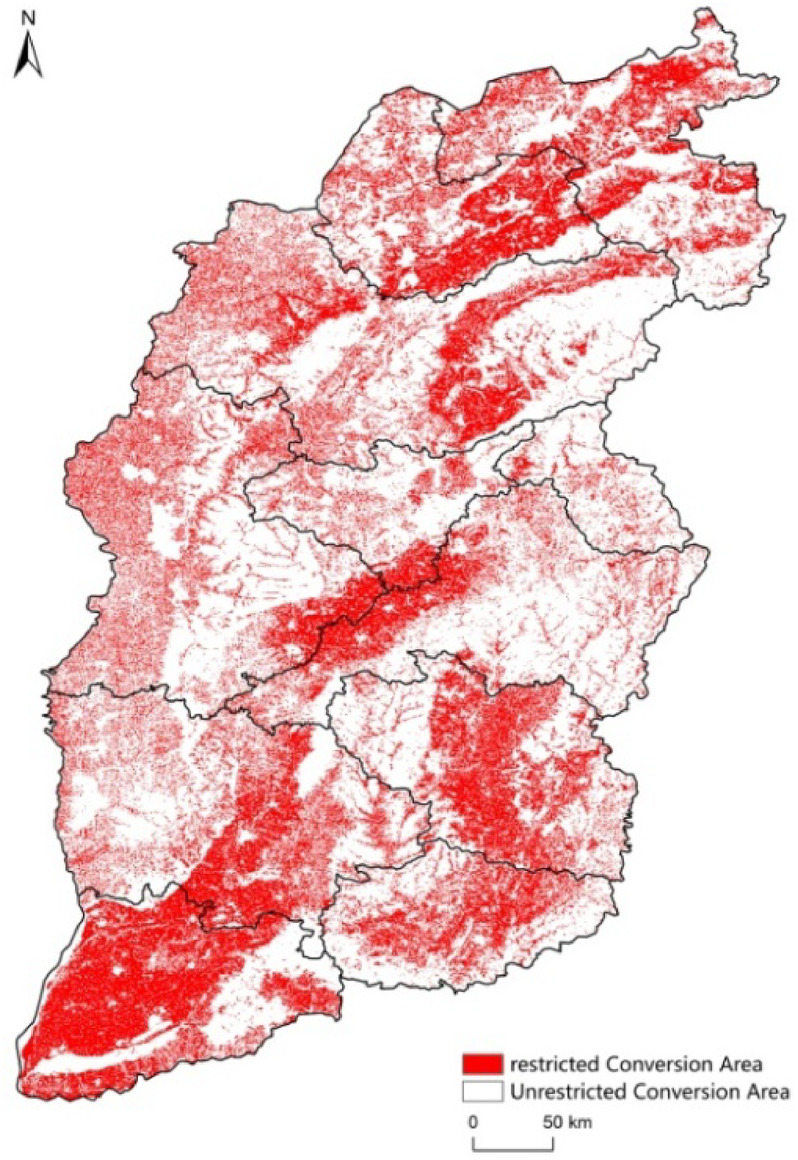
Ecologically sensitive restricted conversion area.

The purpose of this scenario is to verify the impact of arable land protection policies on the ecosystem, realize the dual control of arable land quantity and quality through compulsory intervention, and explore the relationship between food security and ecological protection. Docking the country’s strategy of “strictly adhering to the red line of 1.8 billion mu of arable land” and implementing the balance clause. The conversion probability of arable land to construction land is reduced by 70%; the conversion probability of construction land to arable land is increased by 30%; the conversion probability of unused land to arable land is increased by 50%; and the Fenhe River Valley, basin plain and other areas are designated as stable arable land protection areas(Fig. 4). In this scenario, the “compensation mechanism for cultivated land occupation” is specially introduced, which requires that the new construction land should be balanced by means of reclamation of abandoned mines and improvement of saline-alkali land. This scenario verifies the positive regulatory effect of arable land protection policies on regional ecological security, and quantifies the conservation potential of ecosystem service value under the constraint of arable land red line^43^.

Scenario 3: Accelerated Economic Development Scenario (AEDS).

This scenario aims to simulate the extreme expansion mode of land use under the background of coal resource-based economic transformation, quantify the impact threshold of rapid industrialization on the ecosystem, and warn the risk of ecological protection red line breakthrough. In response to the layout of the central urban agglomeration in Shanxi Province’s *“Land and Space Planning of Shanxi Province (2021–2035)”*^44^, the failure scenario of the implementation of the *“ecological protection red line supervision method”* was simulated^45^. By setting the probability of forest and grassland conversion to construction land increased by 30%, the probability of construction land conversion to arable land, forest, grassland and water decreased by 40%, and the probability of unused land conversion to construction land increased by 50%, the rapid industrialization process of economic hot spots such as urban agglomeration in central Shanxi and the economic belt along the Yellow River was simulated. This scenario reveals the potential loss threshold of ecosystem services under the disorderly development mode, and provides counterfactual evidence for the development of a dynamic adjustment mechanism for the ecological protection red line^46^.

#### Temporal variation characteristics of land use

**Fig. 5:**
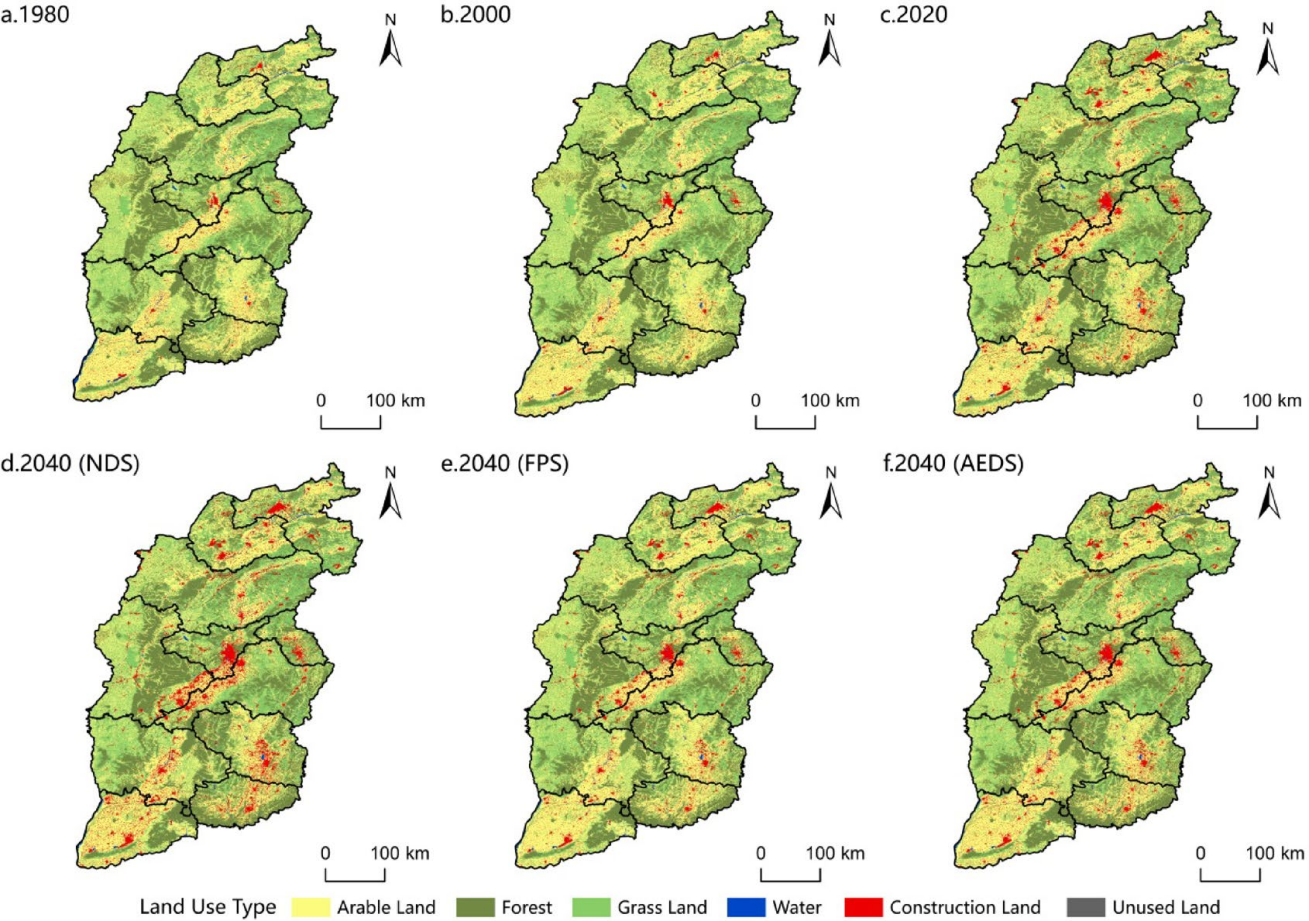
Land use change and simulation forecasting in Shanxi Province.

During the study period, the proportion of arable land in Shanxi Province was the highest, which was the dominant land type, followed by grassland and forest, and the proportion of water, construction land and unused land was small(Fig. 5).

From 1980 to 2020, the arable land decreased by 357,374 ha, reaching 5,780,687 ha, a decrease of 5.82%; the forest increased by 44,480 ha, reaching 4,437,070 ha, an increase of 1.01%; the grassland decreased by 145,836 hectares, reaching 4,426,524 ha, a decrease of 3.19%; the water decreased by 28,652 hectares, reaching 147,680 ha, a decrease of 16.25%; the construction land increased by 490,603 ha, reaching 863,010 ha, an increase of 131.74%; the unused land decreased by 3742 ha, reaching 10,870 ha, a decrease of 25.61%. On the whole, the growth rate of construction land is the fastest (131.74%); except for forest, the rest of the land types were reduced, and the unused land decreased the fastest (26.61%).

By 2040(NDS): the arable land is expected to decrease by 243,081 ha, reaching 5,537,606 ha, a reduction of 4.21%; the forest is anticipated to increase by 52,199 ha, totaling 4,489,269 ha, an expansion of 1.18%; the grassland is likely to decrease by 131,473 ha, to 4,295,050 ha, a decline of 2.97%; the water are projected to increase by 6223 ha, reaching 153,903 ha, a growth of 4.21%; the construction land is expected to rise significantly by 318,416 ha, to 1,181,427 ha, a substantial increase of 36.90%; the unused land is likely to decrease by 2285 ha, to 8586 ha, a reduction of 21.02%.

By 2040(FPS): the arable land is anticipated to increase by 251,279 ha, reaching 6,031,966 ha, an increase of 4.35%; the forest is anticipated to increase by 8259 ha, reaching 4,445,329 ha, an increase of 0.19%; the grassland is expected to increase by 18,444 ha, reaching 4,444,968 ha, an increase of 0.42%; the water is expected to increase by 995 ha, reaching 148,675 ha, an increase of 0.67%; the construction land is expected to decrease by 277,738 ha, reaching 585,272 ha, a decrease of 32.26%; the unused land is expected to increase by 1239 ha, reaching 9632 ha, a decrease of 11.39%.

By 2040(AEDS): the arable land is anticipated to decrease by 28,882 ha, reaching 5,751,805 ha, a decrease of 0.50%; the forest is anticipated to decrease by 13,954 ha, reaching 4,423,116 ha, a decrease of 0.31%; the grassland is anticipated to decrease by 28,038 ha, reaching 4,398,486 ha, a decrease of 0.63%; the water is anticipated to increase by 1507 ha, reaching 149,187 ha, an increase of 1.02%; the construction land is anticipated to increase by 69,764 ha to 932,775 ha, an increase of 8.08%; the unused land is anticipated to decrease by 398 ha to 10,473 ha, a decrease of 3.66%.

#### Spatial variation characteristics of land use

**Fig. 6:**
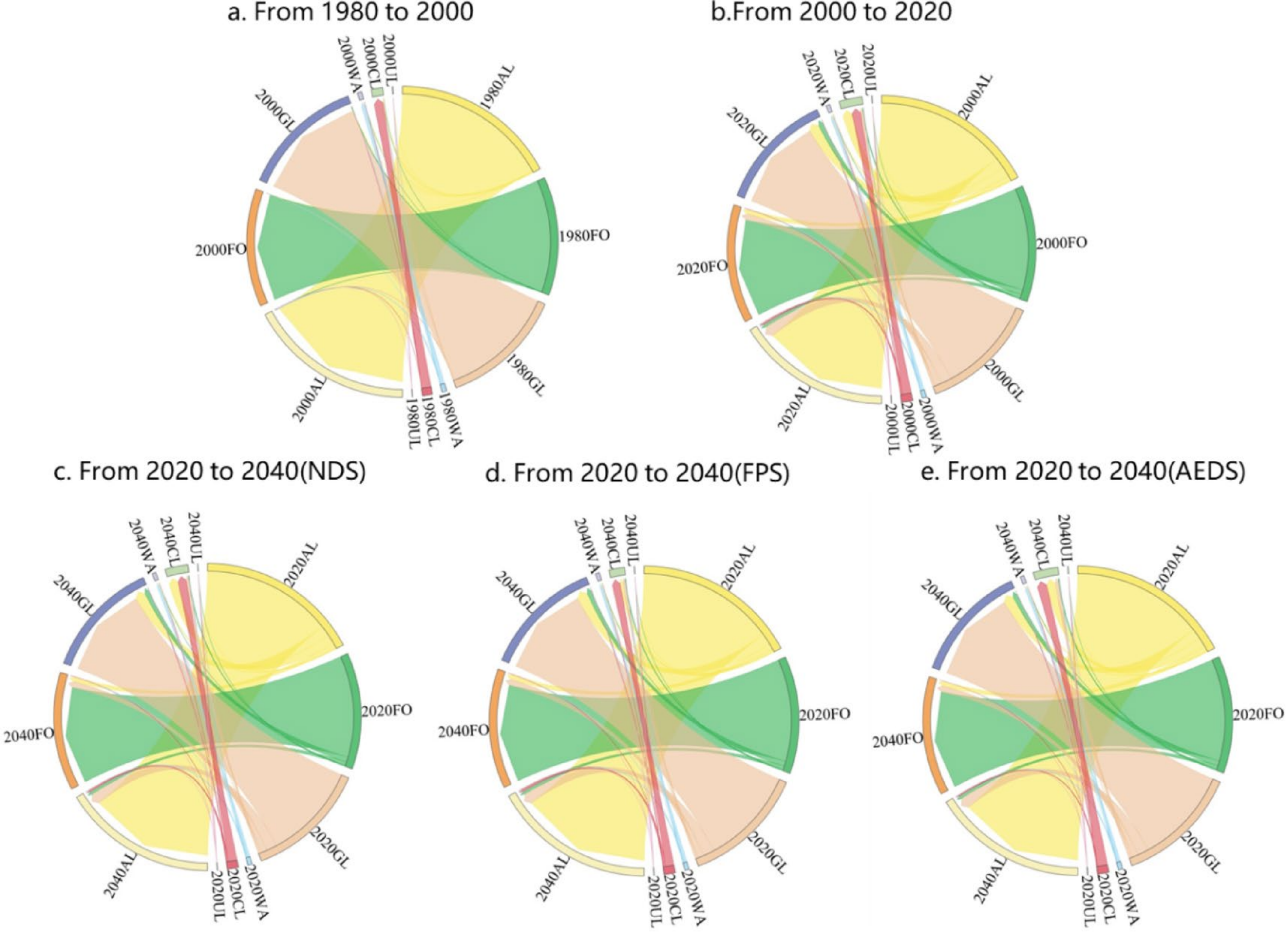
Land use transfer chord diagram.

During the study period, land use transfer was mainly concentrated between arable land, grassland and construction land(Fig. 6).

From 1980 to 2020, 40.23% of the lost arable land was degraded to grassland, mainly distributed in ecologically fragile areas such as Lvliang Mountain, and 38.27% turned to construction land, mainly distributed in plain basins such as Taiyuan Basin. 55.92% of the grassland lost was converted to cultivated land, mainly distributed in the lower slope of the foothills, and 30.32% was converted to forest land. The impact of returning farmland to forest was mainly distributed in the Taihang Mountains, Lvliang Mountains and other mountain areas.

From 2020 to 2040(NDS), 41.86% of the lost arable land will be degraded to grassland, and 36.25% will be turned to construction land; 55.66% of the lost grassland was converted to cultivated land, and 30.79% was converted to forest land. A large proportion of the land lost from cultivated land and grassland is turned to construction land to meet the needs of economic and urbanization development, but this may also lead to a decline in the quality of the ecological environment.

From 2020 to 2040 (FPS), 56.45% of the lost arable land will be degraded to grassland, 25.80% will be converted to forest land, and 14% will be converted to construction land; however, 55.66% of the lost grassland was converted to arable land, 30.79% to forest, and 12.33% to construction land. Affected by the protection policy, the land lost from cultivated land is more converted to forest and grassland with higher ecosystem service value. In addition to compensating for the loss of arable land, the land lost from grassland is mainly converted to forest with higher value, thus improving the level of ecological protection in the province.

From 2020 to 2040(AEDS), 41.86% of the lost arable land will be degraded to grassland, and 36.25% will be turned to construction land; however, 53.62% of the lost grassland was converted to arable land, 29.66% to forest, and 15.54% to forest. More than 30% of the land lost from arable land and grassland is converted into construction land to promote economic development and urbanization, but this may also cause greater pressure on the ecological environment.

Overall, from 1980 to 2020, the loss of arable land and grassland showed obvious regional characteristics. From 2020 to 2040, there are significant differences in the direction and proportion of land transfer under different scenarios. The Farmland Protection Scenario(FPS) pays more attention to ecological protection, and the direction of land transfer is conducive to improving the value of ecosystem services. The Natural Development Scenario(NDS) and the Accelerated Economic Development Scenario(AEDS) are more likely to meet the needs of economic development and urbanization, but may lead to a decline in the quality of the ecological environment. Future land use planning needs to seek a balance between economic development and ecological protection.

### Analysis of ecosystem service value in Shanxi Province

#### Calculation of ecosystem service value in Shanxi Province

The average standard equivalent of the study area from 1980 to 2020 was calculated to be 1031.93 yuan/hm^2^ after correction. Based on the correction results, the coefficient table of ecosystem service value per unit area in Shanxi Province was established**(**Table 4), and the vegetation correction coefficient was introduced to calculate the ecosystem service value in Shanxi Province.

**Fig. 7:**
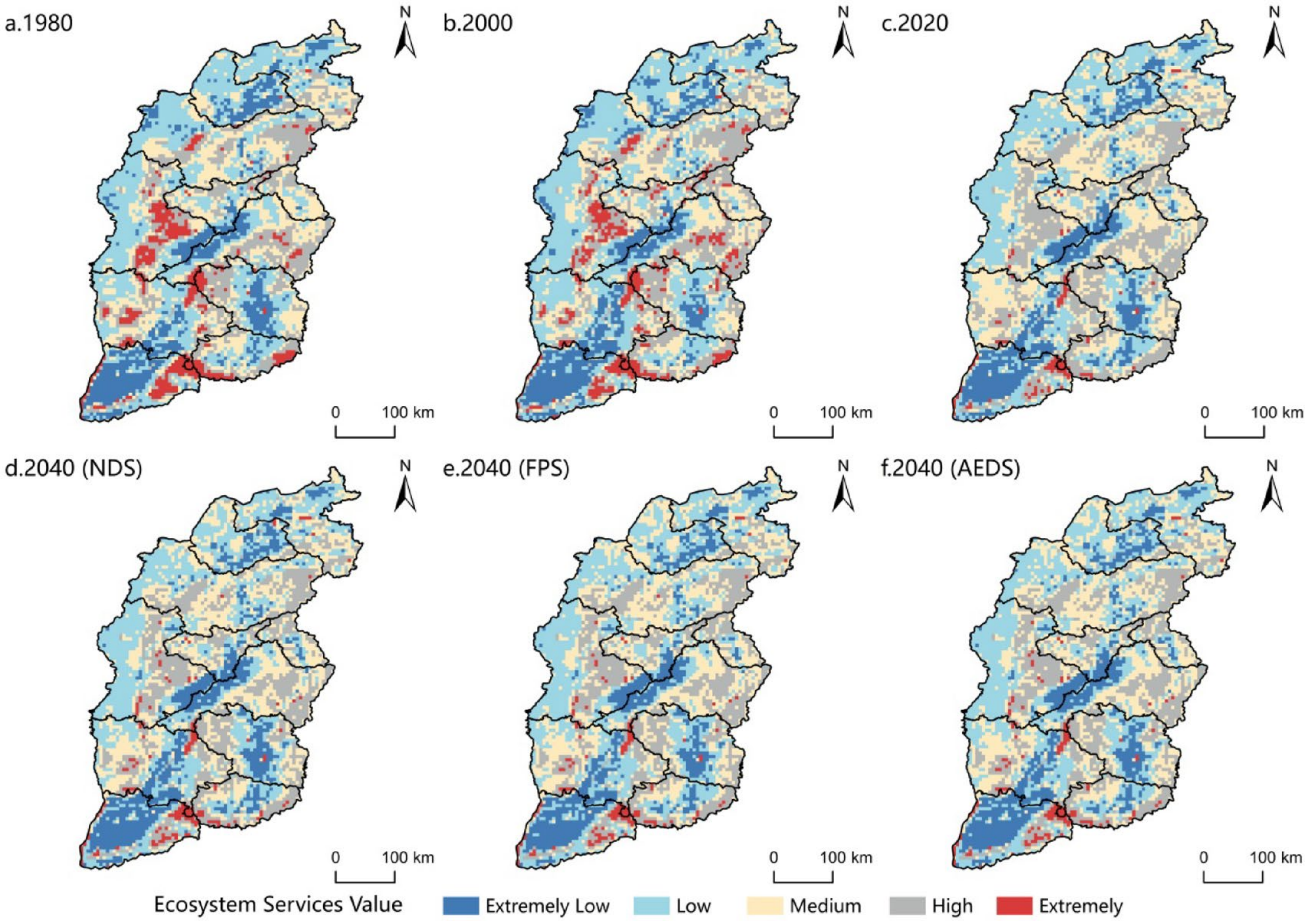
Temporal and spatial changes of ecosystem service value in Shanxi Province.

The extremely high value areas of ecosystem services in the province are mostly located in Taihang Mountains, Lyuliang Mountains and other mountains. The region is rich in natural resources, lush vegetation, and human impact on the natural environment is lighter; the middle value area is mainly distributed in the rivers and lakes such as the Yellow River and the Fenhe River. The terrain is mainly valleys, the terrain is low, and the interference of human activities is weak. The extremely low value area is located in Yuncheng Basin, Linfen Basin and other basin areas. It has flat terrain, large population density and high urbanization level. It is the main population settlement area and economic development area. The land use is mainly cultivated land and construction land, which has a great impact on the natural environment. (Fig. 7)

**Table 4:** Coefficient of ecosystem service value per unit area of each region.

Typology	Arable land	Forest land	Grassland	Water areas	Construction land,	Unused land
FP	877.14	319.90	392.13	825.54	0.00	0.00
MP	412.77	732.67	577.88	237.34	0.00	0.00
WS	20.64	381.81	319.90	8554.70	0.00	0.00
GR	691.39	2425.04	2032.90	794.59	0.00	20.64
CR	371.49	7254.47	5376.36	2363.12	0.00	0.00
AP	103.19	2053.54	1774.92	5727.21	0.00	103.19
WR	278.62	3622.07	3941.97	105504.52	0.00	30.96
SR	1062.89	2951.32	2476.63	959.69	0.00	20.64
NC	123.83	227.02	185.75	72.24	0.00	0.00
BM	134.15	2683.02	2249.61	2631.42	0.00	20.64
AL	61.92	1176.40	990.65	1950.35	0.00	10.32
Total	4138.03	23827.26	20318.70	129620.72	0.00	206.39

Note: FP: Food Production MP: Material Production WS: Water Supply GR: Gas Regulation CR: Climate Regulation AP: Air purification WR: Water Regulation SR: Soil Retention NC: Nutrient Cycling BM: Biodiversity Maintenance AL: Aesthetic Landscape.

From 1980 to 2020, the value of ecosystem services in the province showed a downward trend as a whole, but showed a trend of increasing first and then decreasing in some areas. The value of ecosystem services decreased from 251.054 billion yuan to 245.899 billion yuan, a decrease of 5.155 billion yuan, a decrease of 2.05%. Among them, from 1980 to 2000, the value of ecosystem services increased from 251.054 billion yuan to 254.064 billion yuan, an increase of 3.01 billion yuan, an increase of 1.2%; from 2000 to 2020, the value of ecosystem services decreased from 254.064 billion yuan to 245.899 billion yuan, a decrease of 8.165 billion yuan, a decrease of 3.21%.

By 2040(NDS), the value of ecosystem services in the province will be reduced to 244.811 billion yuan, a decrease of 1.088 billion yuan from 2020, a decrease of 0.44%; by 2040(FPS), the value of ecosystem services in the province will increase to 247.975 billion yuan, an increase of 2.076 billion yuan from 2020, an increase of 0.84%; by 2040(AEDS), the value of ecosystem services in the province will decrease to 245.497 billion yuan, a decrease of 402 million yuan compared with 2020, a decrease of 0.16%.

#### Contribution of land use change to ecosystem service value

**Fig. 8:**
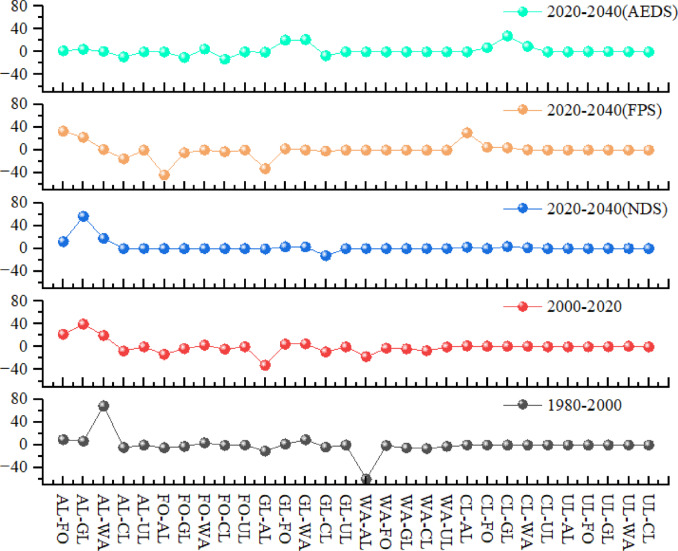
The contribution of land use change to the change of ecosystem service value.

Through the analysis of the contribution of land use change to ecosystem service value in Shanxi Province, the impact of land use change on ecosystem service value is further discussed(Fig. 8). In the five periods of 1980–2020, 2000–2020, 2020–2040(NDS), 2020–2040 (FPS) and 2020–2040 (AEDS), the largest gains in ecosystem service value were AL-WA(68.5265%), AL-GL(39.6850%), AL-GL (56.2967%), AL-FO (33.1166%) and CL-GL(27.7317%). The largest reduction in ecosystem service value was WA-AL(59.5094%), GL-AL(32.5845%), GL-CL(12.4238%), FO-AL(43.3101%) and FO-CL (13.0283%).

The increase and decrease of ecosystem service value are mainly concentrated between cultivated land, forest land, grassland and water area, which is mainly due to the change of value caused by the implementation of the policy of returning farmland to forest and grassland and the replacement between cultivated land, forest land and grassland, but its proportion, is decreasing. The value of ecosystem services generated by the conversion of construction land to other land types is increasing, and the value of ecosystem services generated by the conversion of other land types to construction land is decreasing. This is the result of mutual conversion between land types, but the final value of ecosystem services is mainly affected by the difference between land types and vegetation coefficient.

**Fig. 9:**
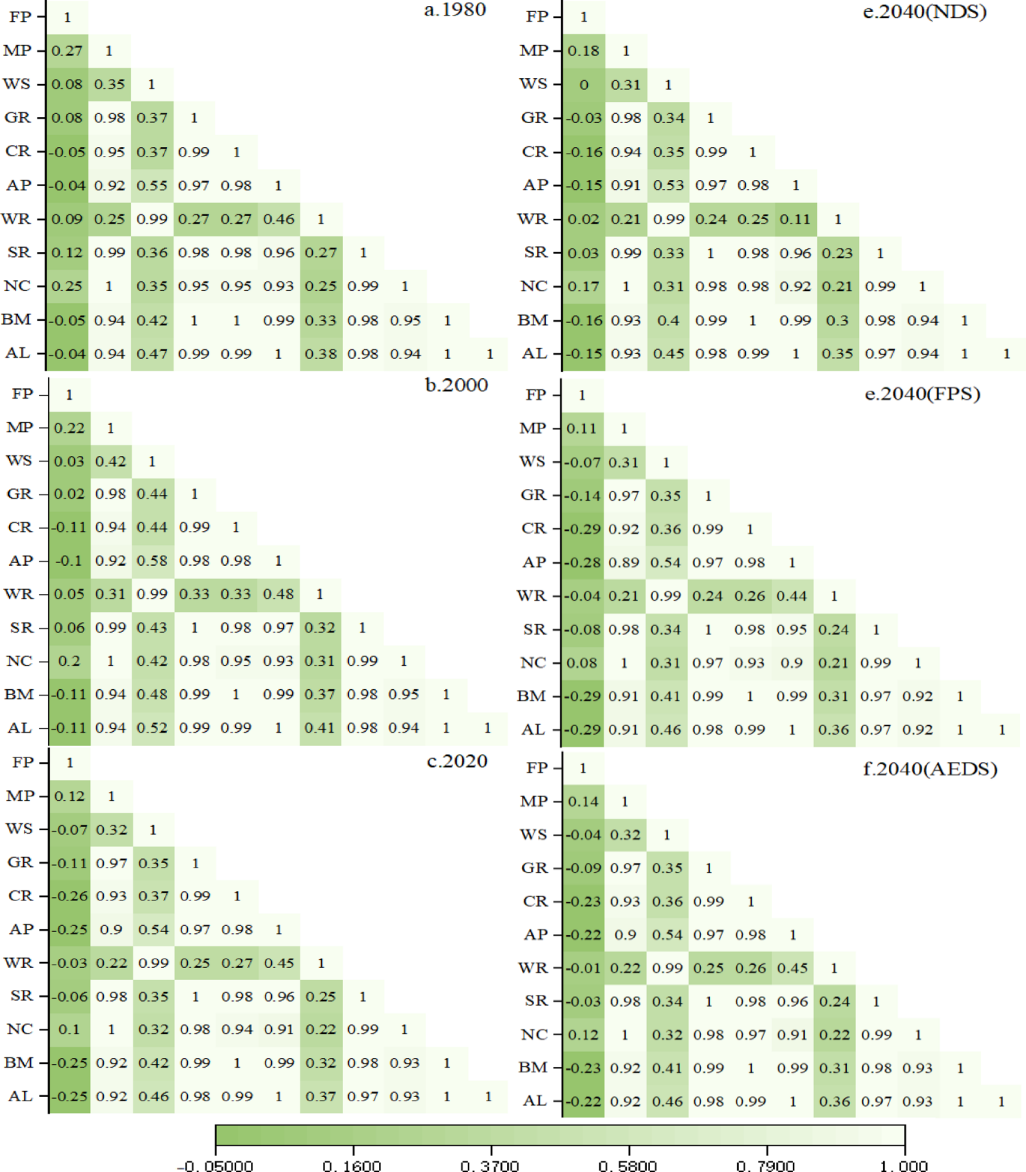
Ecosystem services value tradeoffs synergies.

### Trade-off synergistic effect of ecosystem service

Through pearson correlation analysis of 1980,2000,2020,2040(NDS), 2040(FPS) and 2040(AEDS), a total of 330 groups of correlations were formed, including 293 groups of positive correlations and 37 groups of negative correlations. The trade-off relationship accounted for 11.21%, the synergistic relationship accounted for 88.79%, and the strong synergistic relationship accounted for 52.73%, that is, the synergistic relationship was the main body of the correlation of ecosystem service value in the province (Fig. 9).

From 1980 to 2020, the trade-off intensity (|r|) of FP-CR, FP-EP, FP-BM and FP-PL gradually increased, and four trade-off relationships of FP-WS, FP-GR, FP-WR and FP-SC were added, indicating that agricultural activities had an important impact on ecosystem service functions, and the conflict with ecological protection gradually intensified.

In 2040(FPS), compared with 2020, the trade-off intensity (|r|) will further increase, and agricultural activities will reduce other ecosystem service functions, which will further aggravate the conflict with ecological protection. In 2040(AEDS) and 2040(NDS), the trade-off intensity (|r|) and the trade-off relationship decreased, indicating that the impact of agricultural activities on ecosystem functions gradually decreased under the Natural Development Scenario, and the conflict with ecological protection was alleviated. In general, the changes in the intensity of agricultural production activities and the number of arable land in the province will lead to the reduction of other ecosystem service functions, but the trade-off relationship is still the main body under whatever scenario simulation, and most of the ecosystem service functions are mutually reinforcing.

### Analysis of ecosystem service bundles

#### Identification of ecosystem service bundles types

Bundle analysis can divide units with high similarity into the same ecosystem service cluster. In this paper, K-Means bundle analysis method is selected. The specific calculation formula is detailed in reference^47^.

**Fig. 10:**
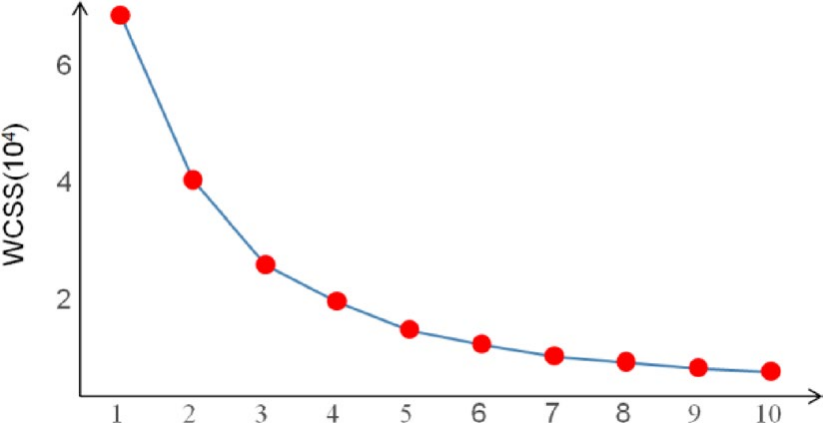
The optimal clustering number determined.

In order to avoid the subjectivity of determining the number of bundles in the K-Means algorithm, this paper uses the elbow method to determine the optimal number of bundles. As shown in the Fig. 10, when the clustering number is 3, the change range tends to be gentle, so the optimal clustering number is 3.

**Fig. 11:**
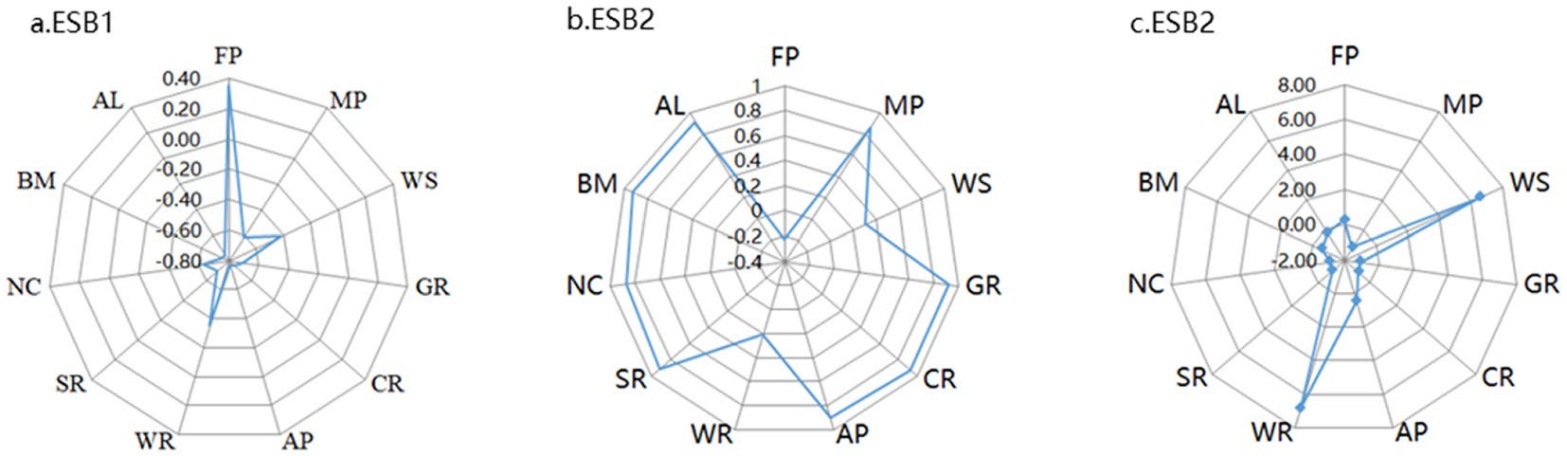
Functional structure of ESBs in Shanxi Province.

Through the Kohonen and Readxl package in the R language, the bundle analysis of the value of ecosystem services in Shanxi Province was realized^48^, and a total of three ecosystem service bundles were generated (Fig. 11).

**Fig. 12:**
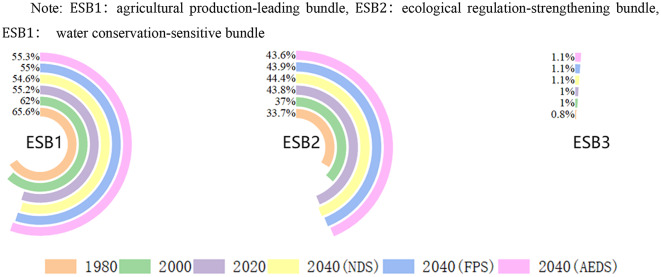
Changes in the proportion of ESB.

**Fig. 13:**
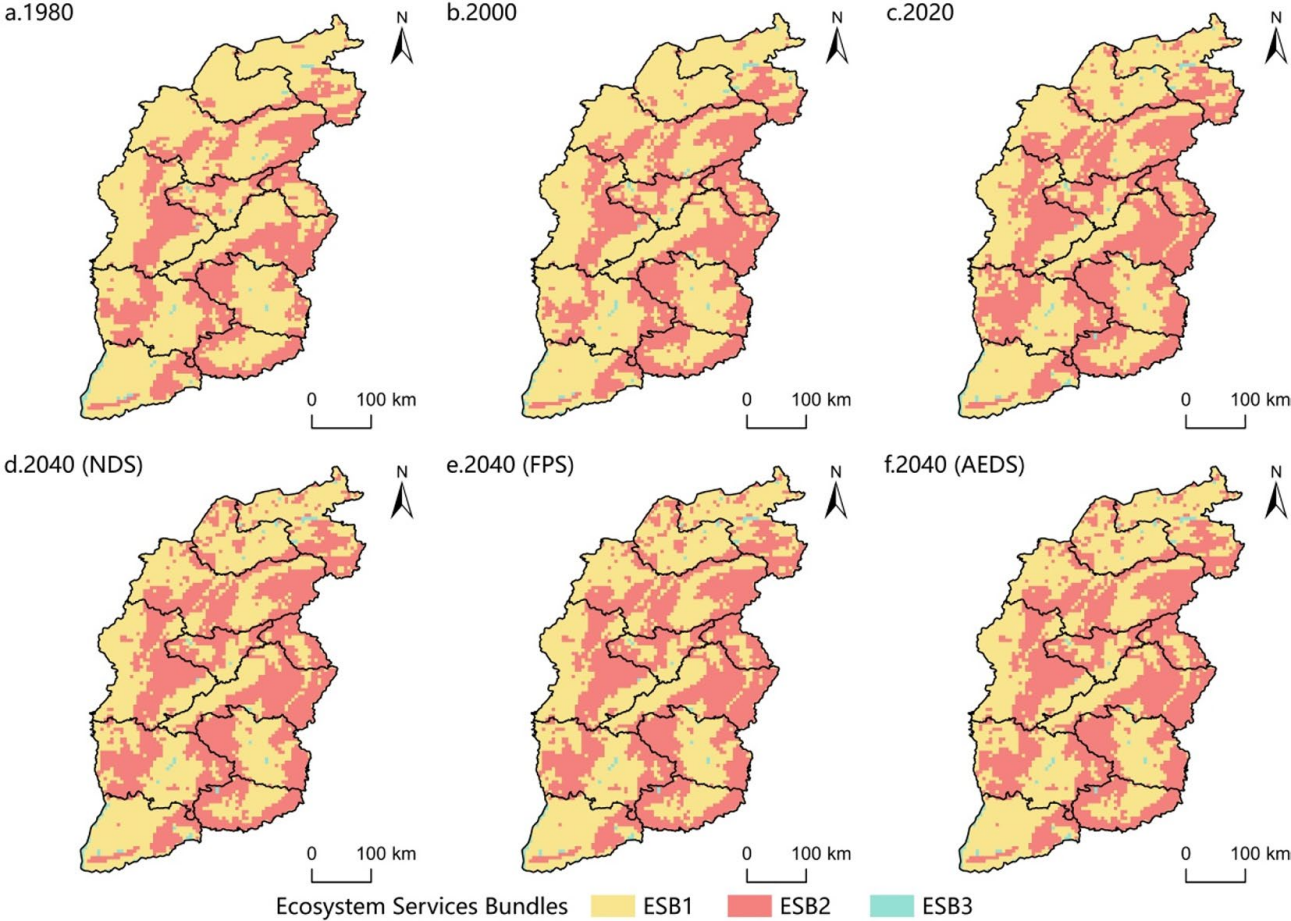
Spatial distribution of ESBs in Shanxi Province.

The ecological function of ESB1 is mainly FP, which is mainly distributed in the basin area. The land types are mainly cultivated land and construction land, and the proportion is decreasing. The ecological functions of ESB2 are mainly EP, BM and PL, mainly distributed in Taihang Mountains, Lyuliang Mountains and other mountains. The land types are mainly woodland and grassland, and the proportion is on the rise, and the rising speed is fast. The ecological functions of ESB3 are mainly WP and HR, which are mainly distributed along the Yellow River, rivers and lakes. The land type is mainly water area, and the proportion is on the rise, and the rising speed is slow(Fig. 12, Fig. 13).

Overall, ESB1 was the main ecosystem service bundle in Shanxi Province at any time, but the proportion showed a downward trend; ESB2, showing an upward trend and rising faster, is becoming more and more important.

#### Evolutionary trajectory analysis of ecosystem service bundles

Based on the clustering results of K-Means value, the stable mapping change trajectory method was used to explore the evolution trajectory of ecosystem service bundles in three time lines of 1980-2000-2020-2040(NDS), 1980-2000-2020-2040 (FPS) and 1980-2000-2020-2040 (AEDS).

According to the stable mapping change trajectory analysis method proposed by Swetnam^49^, combined with the actual situation, this paper proposes a STD determination method (Table 5) suitable for the change trajectory of ecosystem service clusters in Shanxi Province. The change trajectory types are divided into five types: stable type, metastable type, gradual type, cyclic type and other type.

**Table 5:** STD determination principle of ESBs evolution trajectory.

	Stable type	Metastable type	Gradual type	Cyclic type	Other type
Number of changes	0	1	1	2	2
Diversity	1	2	2	2	2、3
similarity	4	3	2	3	1、2
Typical example	1111	1113、2111	1122、1133	1211、3233	1233、2133

Note: Codes 1,2 and 3 represent ESB1, ESB2 and ESB3. Stable Type: The ESB type remains unchanged at all four time nodes; Sub-Stable Type: The ESB type remains unchanged at all three time points and changes at one time point. Gradual Type: The ESB type gradually changes at four time nodes. Cyclic Type: The ESB type changes cyclically between two time nodes. Other Type: The change of ESB type is complex and does not conform to the above types.

Under the three simulation scenarios, the proportion of stable type accounted for more than 82.6%, forming an absolute dominant model (Table 6). This confirms that there is a strong synergistic relationship between land use types and ecosystem service functions in Shanxi Province. It is worth noting that the proportion of stable type reached a peak (82.91%) under the economic priority scenario, suggesting that economic activities may strengthen the ecological function lock-in effect in some regions through intensive land use.

The metastable type (6.84–7.16%) and the gradual type (6.07–6.26%) constitute the secondary evolution model, and their numerical fluctuations show obvious scenario sensitivity. Under the Farmland Protection Scenario, the proportion of metastable ecosystem decreased slightly (7.12%) and increased cyclically (3.61%), reflecting that agricultural policy intervention may break the metastable balance of the original ecosystem and promote some regions to enter the game cycle of “cultivated land-ecology”. The proportion of gradual change in the Accelerated Economic Development Scenario rises to 6.26%, suggesting that there is a gradual land use transformation driven by the market mechanism, and this quantitative change accumulation may constitute a risk point for future qualitative change.

In the three scenarios, the cyclic type (3.43–3.61%) and other types (0.52–0.56%) total less than 4.2%, indicating that the whole system is in a state of strong dissipative structure. The low proportion of nonlinear evolution trajectories confirms that the resilience threshold of the Loess Plateau ecosystem is high, which may be related to the characteristics of ecological resilience under arid and semi-arid climate conditions. However, it is worth noting that the proportion of other types in the natural development scenario is 0.54%, which is higher than that in the planning scenario (FPS 0.52%, AEDS 0.56%), suggesting that human active intervention may compress the abnormal fluctuation space of the ecosystem.

**Table 6:** Area proportion of ESB evolution type under different scenarios (unit:%).

Type of evolutionary trajectory	NDS	FPS	AEDS
stable type	82.60	82.69	82.91
metastable type	7.16	7.12	6.84
gradual type	6.19	6.07	6.26
cyclical type	3.51	3.61	3.43
other type	0.54	0.52	0.56

### Analysis of driving factors of ecosystem service bundle

Based on the results of K-Means bundle, the influencing factors of the formation of ecosystem service bundles in Shanxi Province were analyzed by using geographic detectors. In this paper, considering the geographical and topographic characteristics of Shanxi Province, five natural environmental factors and four social and economic factors are selected to construct a driving factor index system that affects the spatial differentiation of ecosystem service clusters in Shanxi Province. The geographical detector was used to detect and identify the driving factors affecting the spatial differentiation of ecosystem service bundles in the province. By calculating the variance inflation factor (VIF), the degree of collinearity between all explanatory variables was systematically evaluated. The VIF values are all less than 5 (Table 7), and the condition numbers are all less than 30, indicating that there is no serious collinearity problem between variables.

**Table 7:** The results of collinearity test of driving factors.

	X1	X2	X3	X4	X5	X6	X7	X8	X9
VIF	1.3108	1.2972	1.1505	1.7549	3.6368	1.3125	1.1276	1.2878	1.1890

Note: x1:DEM x2:Slope x3: distance from the river x4: average annual temperature x5: annual precipitation x6: distance from the highway x7: distance from the railway x8: population density x9: GDP.

**Fig. 14:**
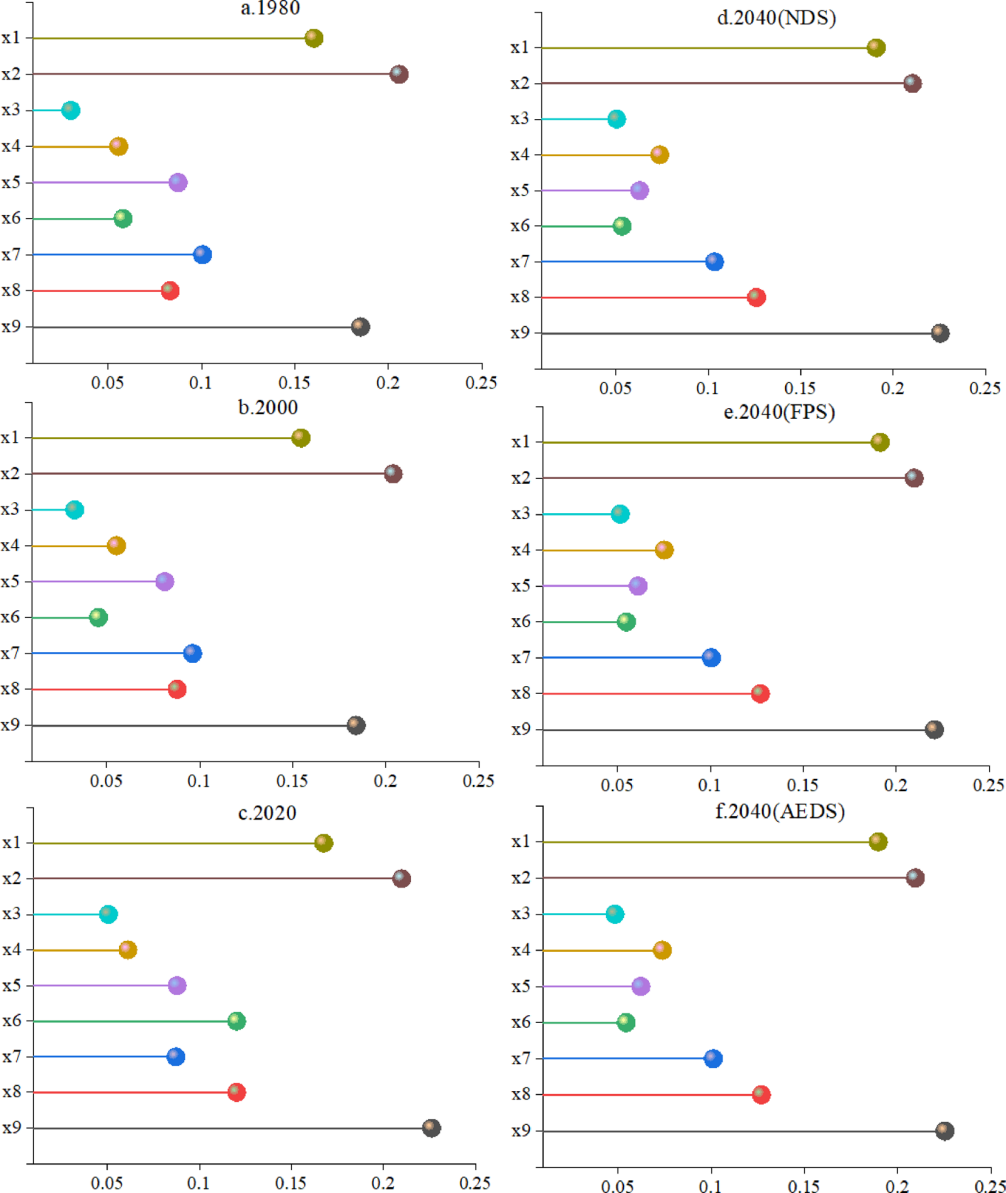
Factor detection results.

Slope and GDP in different periods have an important impact on the distribution of ecosystem service clusters in Shanxi Province (Fig. 14). In 1980, the explanatory power of each driving factor was ranked as follows: slope > GDP > elevation > distance from railway > annual precipitation > population density > distance from highway > annual average temperature > distance from river. In 2000, the explanatory power of each driving factor was ranked as follows: slope > GDP > elevation > distance from railway > population density > annual precipitation > annual average temperature > distance from highway > distance from river. In 2020, the explanatory power of each driving factor was ranked as follows: GDP > slope > elevation > population density > annual precipitation > distance from railway > annual average temperature > distance from highway > distance from river. In 2040 (NDS), the explanatory power of each driving factor was ranked as follows: GDP > slope > elevation > population density > distance from railway > average annual temperature > annual precipitation > distance from highway > distance from river. In 2040 (FPS), the explanatory power of each driving factor was ranked as follows: GDP > slope > elevation > population density > distance from railway > average annual temperature > annual precipitation > distance from highway > elevation > population density > annual precipitation > distance from railway > annual average temperature > distance from highway > distance from river. In 2040 (NDS), the explanatory power of each driving factor was ranked as follows: GDP > slope > elevation > population density > distance from railway > average annual temperature > annual precipitation > distance from highway > distance from river. In 2040 (FPS), the explanatory power of each driving factor was ranked as follows: GDP > slope > elevation > population density > distance from railway > average annual temperature > annual precipitation > distance from highway > distance from river. In 2040 (AEDS), the explanatory power of each driving factor was ranked as follows: GDP > slope > elevation > population density > distance from railway > average annual temperature > annual precipitation > distance from highway > distance from river.

In 1980 and 2000, the slope had the highest explanatory power for the province’s ecosystem service bundles, but in 2020 and 2040, the explanatory power of the slope gradually decreased, and the explanatory power of GDP to the province’s ecosystem service bundles became the highest, indicating that the impact of GDP on ecosystem service bundles is becoming more and more important.

**Fig. 15:**
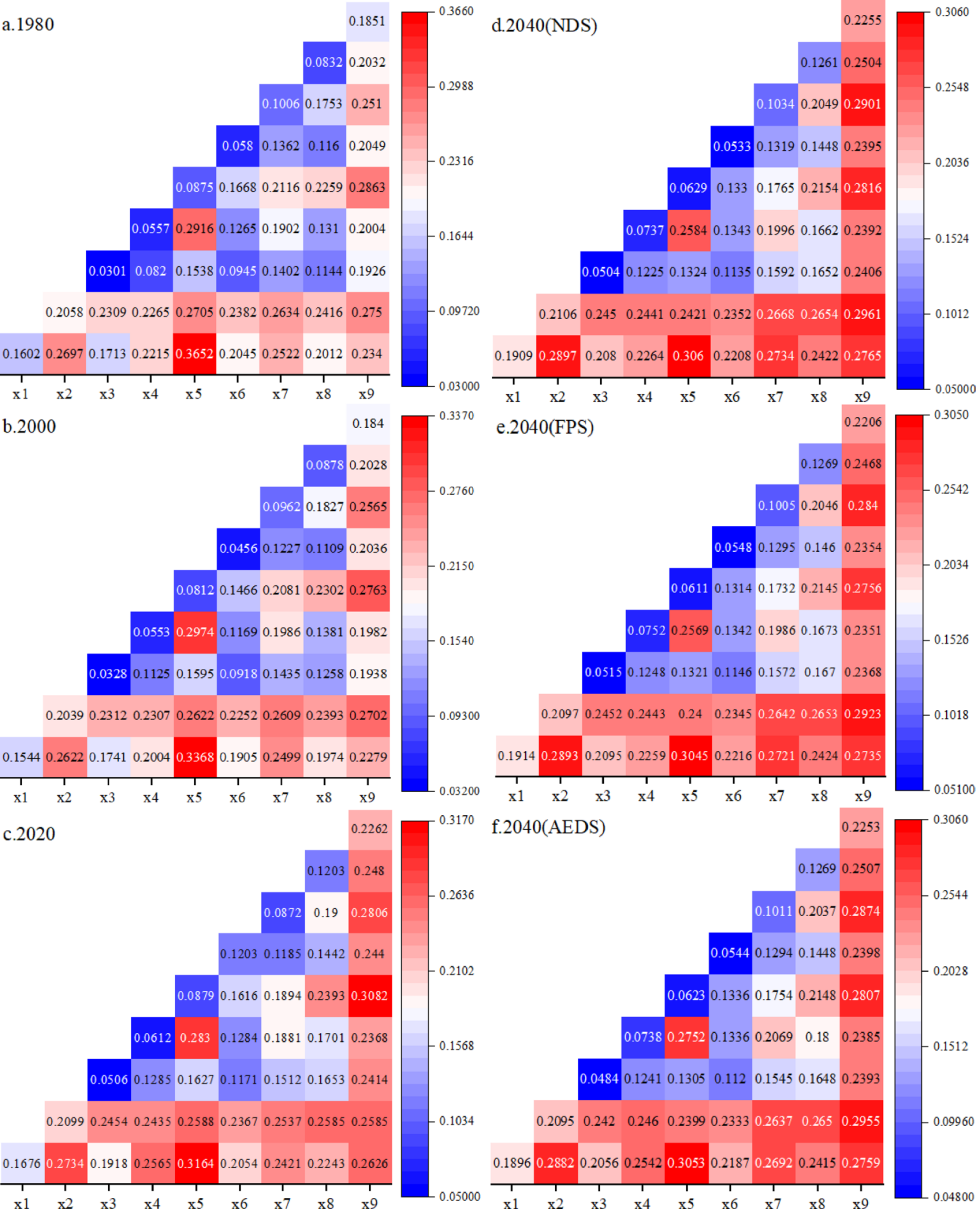
Interactive detection of influencing factors of ecosystem service bundles in Shanxi Province.

In order to explore the influence of the interaction between factors on the distribution of ecosystem service bundles in the province, this paper uses the interaction factor detector to detect the interaction between factors (Fig. 15). The results of the interactive detection of ecosystem service bundles in the whole province are as shown in the figure, and the interaction shows certain regularity under 1980, 2000, 2020, 2040(NDS), 2040(FPS) and 2040(AEDS). In general, the interaction between GDP and slope, distance to river, annual precipitation, distance to highway, distance to railway and population density has a high explanatory power, indicating that GDP has an important impact on the spatial distribution of ecosystem service bundles in the province. In 1980, 2000 and 2020, the interaction between GDP and annual precipitation had the highest explanatory power, which was 0.2863, 0.2763 and 0.3082, respectively. In the three scenarios of 2040, the interaction between GDP and distance from the railway has the highest explanatory power, which is 0.2901, 0.284 and 0.2874, respectively, indicating that the impact of socio-economic factors on the spatial distribution of ecosystem service bundles in the province is gradually increasing, which is consistent with the results of single factor detection.

## Discussion

This study constructs a framework for land use simulation, dynamic ecosystem service value assessment, ecosystem service bundle identification and driving factor analysis, and provides a new paradigm for ecologically fragile areas. Compared with Wang Yupeng et al.^50^.and Lyu Feinan et al.^51^. research on ecosystem service bundles in Shanxi Province, this paper uses a dynamic ecosystem service value assessment model to better quantify changes in ecosystem services, and makes up for the gaps in multi-scenario simulation of ecosystem service bundles in the study area. However, this study did not include the long-term effects of climate change and major projects. Subsequent research will extend the simulation period to 2050, couple climate scenarios with the “double carbon” goal, deepen the research on the resilience mechanism of ESB, and provide decision support for ecological protection and high-quality development in the Yellow River Basin.

The study uses 30-meter resolution data. Although it can accurately describe patch changes, the PLUS model has an error of 12.33% when simulating construction land expansion. In the future, it is necessary to combine hyperspectral data and deep learning algorithms to improve the ability of land conversion identification and further improve the accuracy of ecosystem service value assessment.

Under the Farmland Protection Scenario, the ESB and the stability of ESV is improved, but the conversion rate from grassland to forest is only 30.79%, indicating that natural restoration and artificial restoration need to be combined. Under the Accelerated Economic Development Scenario, the expansion of construction land leads to a slight decrease in ESV, but the proportion of circular ESB increases, suggesting that industrial transformation can be guided by flexible planning to achieve a win-win situation of economy and ecology.

The ecosystem services in Shanxi Province are mainly coordinated, and the contradiction between food production and ecological protection is only manifested in specific service pairs. The synergistic relationship shows that all kinds of ecosystem services in Shanxi generally promote each other, but the FP-CR trade-off coefficient has increased by 23.5% in the past decade, highlighting the conflict between grain production and ecological protection in specific aspects. Follow-up studies will further analyze the relationship between synergy and trade-off.

The spatial heterogeneity of ESV indicates the necessity of differentiated ecological compensation. It is recommended to implement targeted measures in different ESB regions: ESB1 area to implement farmland subsidy and rotation system; the ESB2 area strictly adheres to the ecological protection red line and carries out ecological migration compensation; ESB3 area to establish water quality gambling agreement.

## Conclusion

(1) The land use pattern showed significant stage characteristics.

From 1980 to 2020, the area of arable land in Shanxi Province decreased by 5.82% and the construction land increased by 131.74%. The core driving force comes from the dual role of rapid socio-economic transformation and urbanization strategy. In 2040 (FPS), the arable land increased by 4.35%, which confirmed the constraint effect of cultivated land protection system and requisition-compensation balance policy on land resources. In 2040 (AEDS), construction land expanded by 8.08% but the loss of arable land slowed down, indicating that industrial structure optimization and intensive land use policies can alleviate the contradiction between ecology and development, highlighting the leverage effect of policy tools in regulating land use competition.

(2) Multidimensional driving factors of ecosystem service value fluctuation.

The fluctuation of ESV “N” type is affected by both ecological restoration and human activities. In the early stage, returning farmland to forest and grass increased ESV, and in the later stage, energy development and urbanization led to a decline. Under the Farmland Protection Scenario, ESV rebounded, highlighting the ecological function value of cultivated land. The spatial difference of ESV is determined by topography and human disturbance intensity.

(3) The trade-off relationship of ecosystem services is dominant but the synergistic effect is enhanced.

88.79% of ESV showed a synergistic relationship, and the proportion of strong synergy was more than half (52.73%), indicating that the overall ecosystem services in Shanxi Province showed a synergistic trend. It is worth noting that the trade-off intensity of key service pairs such as FP-CR and FP-EP increases with time, revealing the deep contradiction between food production and ecological protection.

(4) ESB type presents regional functional differentiation and dynamic stability.

Three types of service clusters, namely, agricultural production-leading bundle (ESB1), ecological regulation-strengthening bundle (ESB2), and the water conservation-sensitive bundle (ESB3), were identified, corresponding to the basin agricultural area, mountain ecological area and river and lake wet area, respectively. Under multi-scenario simulation, the proportion of stable ESB exceeded 82%. Under the Accelerated Economic Development Scenario, the proportion of stable type reached the peak (82.91%), suggesting that the market mechanism may strengthen the ecological function lock-in through intensive land use. The spatial distribution of ESB is affected by both natural and socio-economic factors. The slope is dominant in the early stage and the GDP is dominant in the later stage.

## Electronic supplementary material

Below is the link to the electronic supplementary material.

Supplementary Material 1

## Data Availability

The datasets used and/or analysed during the current study available from the corresponding author on reasonable request.
