# Supplementary material for: Identification and analysis of driving factors for ecosystem service bundles in Shanxi Province under multiple scenario simulations
Source: Sci Rep. 2025 Jul 1;15:20789. doi: 10.1038/s41598-025-08876-5 (PMC12219418; doi:10.1038/s41598-025-08876-5)
Supplement: Supplementary file 1 — Supplementary Material 1 [file 41598_2025_8876_MOESM1_ESM.docx]

Identification and analysis of driving factors for ecosystem service bundles in Shanxi Province under multiple scenario simulations

**Guofeng Dang ^a^, Guibin Li ^a ,^*, Jinzhou Hu ^a^**

**^a^****School of Geography and Environmental Sciences,** **Northwest Normal University, Lanzhou 730070，China**

**Corresponding author:** **Guibin Li**

**E-mail address : 3203522964@qq.com**

Supplementary Document

The required parameters and verification indicators of the PLUS model in land use simulation are as follows:

Scenario 1: Natural Development Scenario (NDS)

**Supplementary Table 1** Transition Matrix

|  | Arable Land | Forest | Grass Land | Water | Construction  Land | Unused Land |
| --- | --- | --- | --- | --- | --- | --- |
| Arable Land | 1 | 1 | 1 | 1 | 1 | 1 |
| Forest | 1 | 1 | 1 | 1 | 1 | 1 |
| Grass Land | 1 | 1 | 1 | 1 | 1 | 1 |
| Water | 0 | 0 | 0 | 1 | 0 | 0 |
| Construction Land | 1 | 1 | 1 | 1 | 1 | 1 |
| Unused Land | 1 | 1 | 1 | 1 | 1 | 1 |

**Supplementary Table 2** Neighborhood Weight

|  | Arable Land | Forest | Grass Land | Water | Construction Land | Unused Land |
| --- | --- | --- | --- | --- | --- | --- |
| Weight | 0.9903 | 0.5927 | 0.8533 | 0.0535 | 0.6716 | 0.0000 |

Scenario 2: Farmland Protection Scenario (FPS)

**Supplementary Table 3** Transition Matrix

|  | Arable Land | Forest | Grass Land | Water | Construction  Land | Unused Land |
| --- | --- | --- | --- | --- | --- | --- |
| Arable Land | 1 | 0 | 0 | 0 | 1 | 0 |
| Forest | 1 | 1 | 1 | 1 | 1 | 1 |
| Grass Land | 1 | 1 | 1 | 1 | 1 | 1 |
| Water | 0 | 0 | 0 | 1 | 0 | 0 |
| Construction Land | 1 | 1 | 1 | 1 | 1 | 1 |
| Unused Land | 1 | 1 | 1 | 1 | 1 | 1 |

**Supplementary Table 4** Neighborhood Weight

|  | Arable Land | Forest | Grass Land | Water | Construction Land | Unused Land |
| --- | --- | --- | --- | --- | --- | --- |
| Weight | 0.9898 | 0.6490 | 0.9385 | 0.0460 | 0.3626 | 0.0000 |

Scenario 3: Accelerated Economic Development Scenario (AEDS)

**Supplementary Table 5** Transition Matrix

|  | Arable Land | Forest | Grass Land | Water | Construction  Land | Unused Land |
| --- | --- | --- | --- | --- | --- | --- |
| Arable Land | 1 | 1 | 1 | 1 | 1 | 1 |
| Forest | 1 | 1 | 1 | 1 | 1 | 1 |
| Grass Land | 1 | 1 | 1 | 1 | 1 | 1 |
| Water | 0 | 0 | 0 | 1 | 0 | 0 |
| Construction Land | 1 | 1 | 1 | 1 | 1 | 1 |
| Unused Land | 1 | 1 | 1 | 1 | 1 | 1 |

**Supplementary Table 6** Neighborhood Weight

|  | Arable Land | Forest | Grass Land | Water | Construction Land | Unused Land |
| --- | --- | --- | --- | --- | --- | --- |
| Weight | 0.9891 | 0.6800 | 0.9767 | 0.0603 | 0.8555 | 0.0000 |

Verification Indicators:

Kappa coefficient is 0.78014,

Overall accuracy is 84.82%,
